# Thrombotic Thrombocytopenic Purpura: Revisiting a Miss and an Inevitable Consequence

**DOI:** 10.7759/cureus.9283

**Published:** 2020-07-19

**Authors:** Pooja Gogia, Ezioma Gbujie, Elizabeth Benge, Sidharth Bhasin

**Affiliations:** 1 Internal Medicine, Saint Peter's University Hospital, New Brunswick, USA; 2 Internal Medicine, St. George's University School of Medicine, Bethesda, USA; 3 Internal Medicine, Saint Peter's University Hospital/Rutgers University, New Brunswick, USA

**Keywords:** thrombotic thrombocytopenic purpura, atypical ttp, microangiopathic hemolytic anemia, schistocytes, adamts13

## Abstract

Thrombotic thrombocytopenic purpura (TTP) is typically characterized by the symptomatic pentad of fever, thrombocytopenia, microangiopathic hemolytic anemia, neurologic abnormalities, and renal failure. Atypical TTP is the diagnosis used to describe the subset of patients with TTP who present with symptoms that deviate from the classic pentad.

We report a case an 86-year-old woman who presented to the emergency department complaining of chest pain for one day. She was reportedly on antibiotics for sinus infection. Physical examination revealed multiple bilateral superficial hematomas, predominantly on her extremities. On admission, her lab values were as follows: platelet count of 6,000/cubic millimeter, hemoglobin of 10.4 grams/deciliter, leukocyte count of 5100 cells/cubic millimeter, total bilirubin of 2.3 milligrams/deciliter, and troponin-I of 5.190 nanograms/milliliter. Peripheral blood smear was normal and did not reveal any schistocytes. The patient was admitted to the intensive care unit with a diagnosis of a non-ST-elevation myocardial infarction and a presumed diagnosis of immune thrombocytopenic purpura from antibiotic use. She was treated with intravenous solumedrol and a high-intensity statin. On the third day of her admission, the patient’s mental functioning deteriorated and was intubated to protect her airway. A second peripheral smear revealed schistocytes, and subsequent laboratory studies supported the diagnosis of TTP. Plasma exchange therapy was planned. However, the patient succumbed to cardiac arrest before it could be initiated. The diagnosis was later confirmed with an ADAMTS13 (a disintegrin and metalloproteinase with a thrombospondin type 1 motif, member 13) assay.

This case serves as an example of one of the many ways in which TTP can present, and emphasizes the importance of considering TTP as a differential diagnosis.

## Introduction

Thrombotic thrombocytopenic purpura (TTP) is a rare hematologic disorder caused by unregulated von Willebrand factor (vWF) dependent platelet thrombosis due to decreased activity of ADAMTS13 (a disintegrin and metalloproteinase with a thrombospondin type 1 motif, member 13), which inhibits vWF-dependent platelet aggregation [1,2]. This decrease in enzymatic activity results in massive platelet aggregation and extensive microvascular thrombosis, which leads to persistent microvascular thrombi in multiple organs [3].

TTP has an annual incidence of 3 to 11 cases per million people and an annual prevalence of 10 cases per million people [4]. Characteristic findings include thrombocytopenia with schistocytes on a peripheral blood smear, anemia, renal dysfunction, and an indirect bilirubinemia due to intravascular hemolytic anemia [2]. Symptoms typically include the pentad of fever, thrombocytopenia, microangiopathic hemolytic anemia (MAHA), neurologic abnormalities, and renal failure [1]. “Atypical TTP” is the diagnosis used to describe the subset of patients with TTP who present with symptoms that deviate from the classic pentad [3]. Clinical presentations of TTP can be typical or atypical on initial presentation or upon relapse [3]. Here, we describe a case of atypical TTP that initially presented as a non-ST-elevation myocardial infarction (NSTEMI) with thrombocytopenia, with subsequent development of neurologic deterioration.

## Case presentation

**Figure 1:**
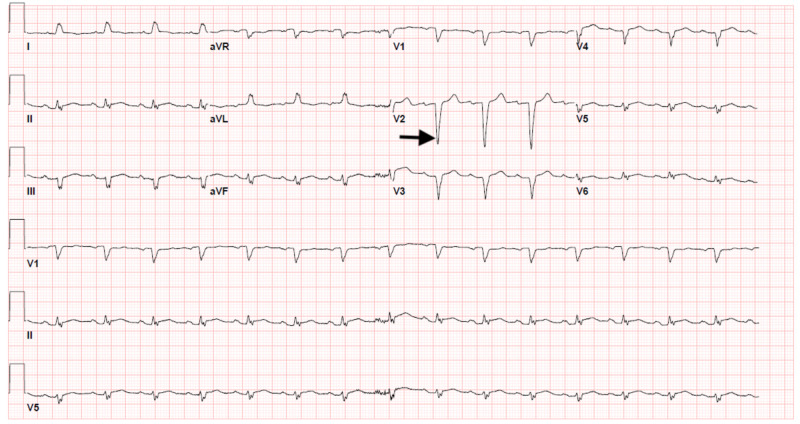
Electrocardiogram showing normal sinus rhythm (atrial and ventricular rate: 93 milliseconds), with a QRS duration of 104 milliseconds. The arrow depicts incomplete right bundle branch block.

**Figure 2:**
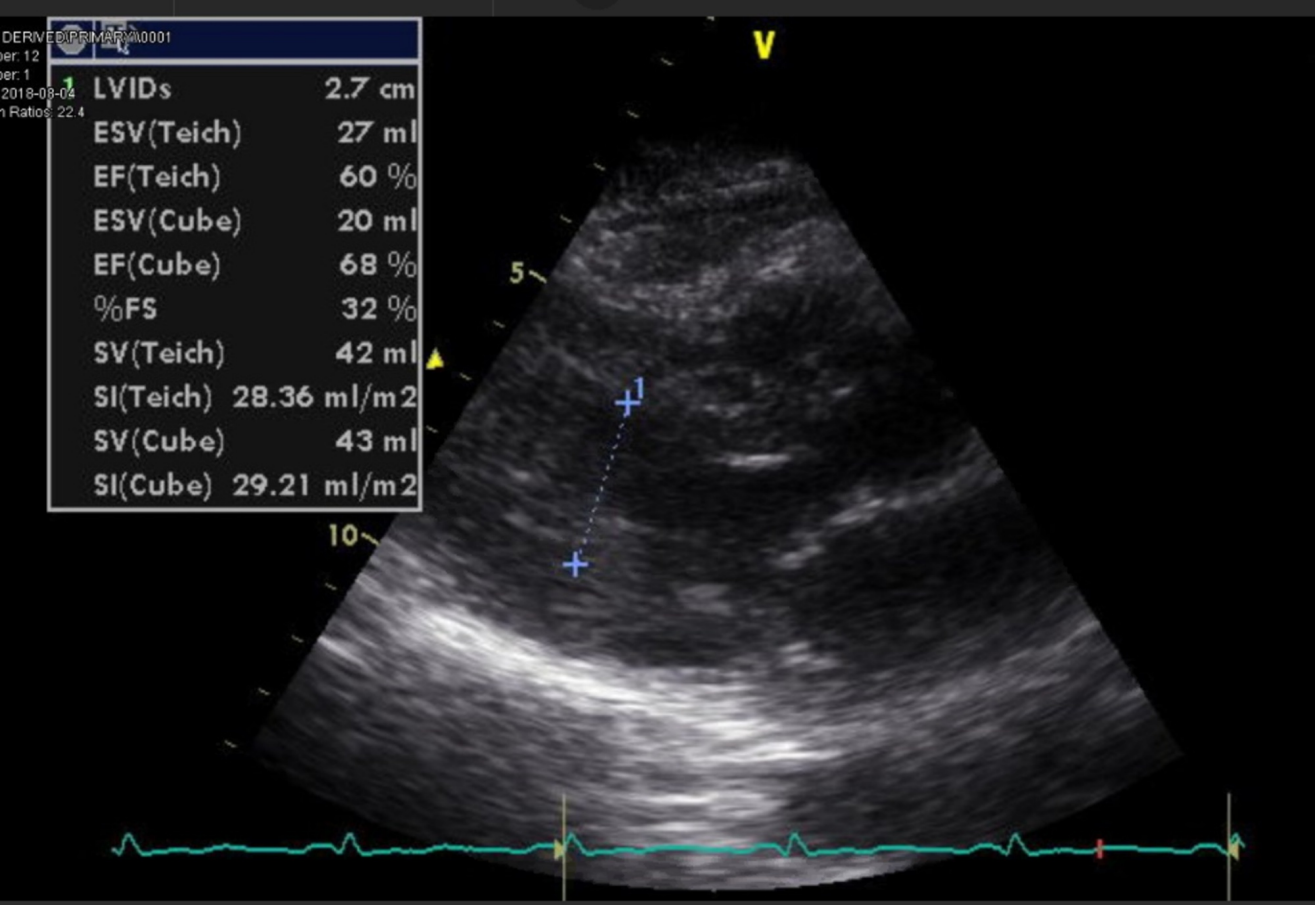
Two-dimensional echocardiogram showing normal biventricular dimensions and left ventricular ejection fraction of 60%.

An 86-year-old female with a past medical history of hypertension presented to the emergency department with chest pressure and left-hand numbness of one-day duration. She was a non-smoker and consumed one glass of wine per day. On presentation, her blood pressure was 167/92 mmHg. Her other vital signs were within normal limits. Physical examination revealed multiple bilateral superficial hematomas, predominantly on her extremities, and mild and bilateral lower extremity pitting edema. Investigations on admission were as follows: platelet count of 6,000/cubic millimeter, hemoglobin of 10.4 grams/deciliter, leukocyte count of 5,100 cells/cubic millimeter, total bilirubin of 2.3 milligrams/deciliter, troponin I of 5.190 nanograms/milliliter, Na of 125 milliequivalent/liter, BUN (blood urea nitrogen) of 23 milligrams/deciliter, and creatinine of 0.72 milligrams/deciliter. Peripheral blood smear was normal. Electrocardiogram showed normal sinus rhythm with incomplete right bundle branch block, as shown in Figure 1. Two-dimensional echocardiogram showed normal biventricular dimensions and systolic function. Left ventricular ejection fraction was 60%, as shown in Figure 2.

The patient was admitted to the intensive care unit (ICU) with a diagnosis of NSTEMI and presumed idiopathic thrombocytopenic purpura. The patient medication history was tablet losartan 50 mg, which she was taking for the last 25 years, and tablet cefuroxime for a sinus infection, which she started taking three days back.

She was started on intravenous (IV) solumedrol and a high-intensity statin. Anti-coagulation therapy was contraindicated at this time. It was decided to medically manage her NSTEMI and trending troponin. She was also started on hypertonic saline due to hyponatremia. Her recent use of cefuroxime, her severe thrombocytopenia, and the lack of schistocytes on peripheral smear supported the diagnosis of immune thrombocytopenic purpura (ITP). The patient’s troponin trended down, but her thrombocytopenia persisted.

**Figure 3:**
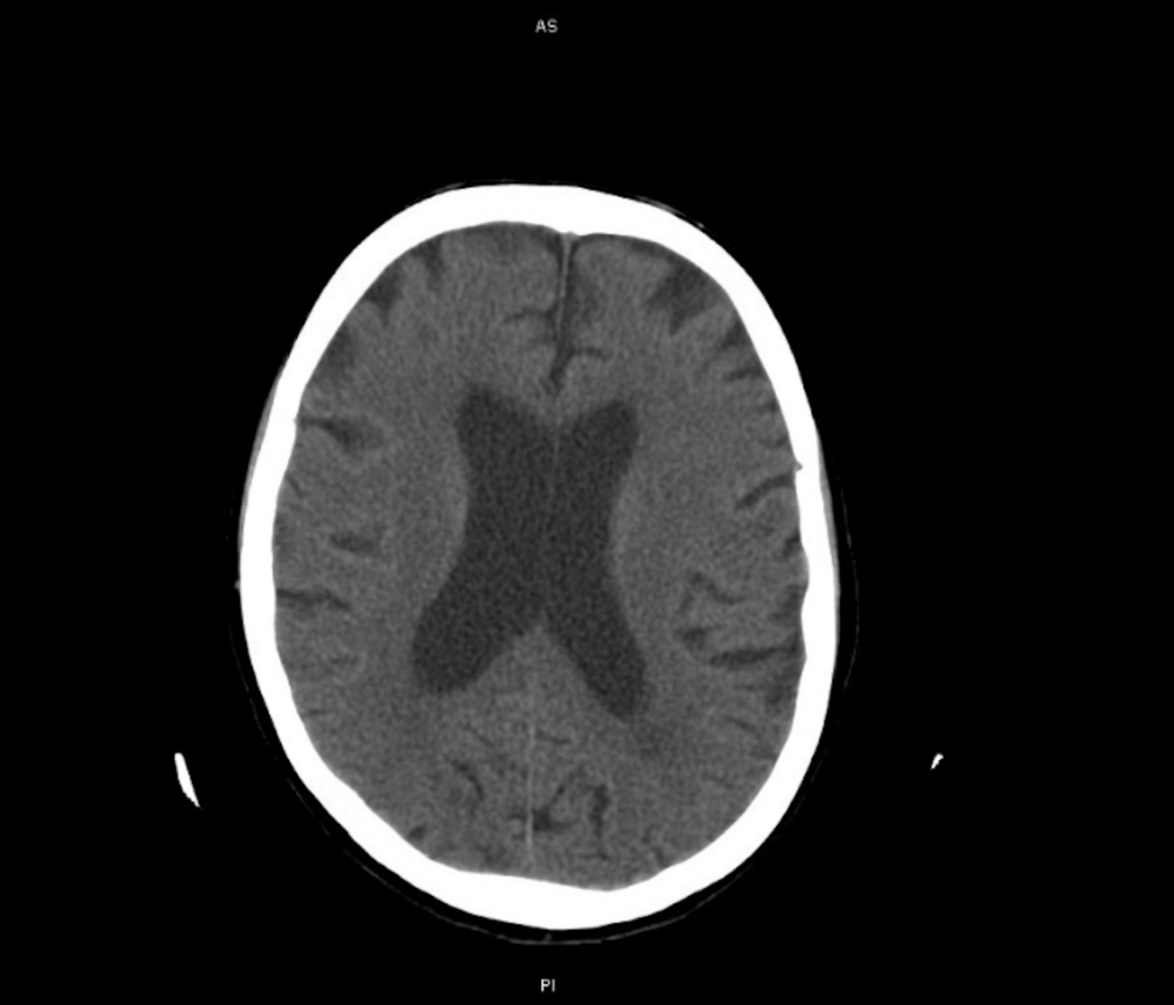
CT demonstrating a normal scan with no acute findings.

On the third day of her admission, the patient experienced a rapid decline in neurologic function. She became increasingly confused and agitated. This decline was initially attributed to a combination of ICU delirium, steroid use, and hyponatremia. A CT scan of the head was performed and was negative for bleeds and acute infarcts, as shown in Figure 3. Her mental status continued to worsen by the hour. She became obtunded within six hours and was intubated to protect her airway.

At this time, TTP was suspected due to the patient’s declining neurologic function. Her lab values from the third day of her hospital admission revealed persistent thrombocytopenia (platelet count of 8,000/cubic millimeter), indirect bilirubin of 2.1 milligrams/deciliter, lactate dehydrogenase of 1,398 units/liters, haptoglobin of <30 milligrams/deciliter, and reticulocyte count of 3.6%, all of which were suggestive of intravascular hemolysis; repeat blood smear revealed schistocytes and hemoglobin of 9.6 milligrams/deciliter. An ADAMTS13 assay was collected at this time.

Plasma exchange therapy was then planned. However, the patient succumbed to cardiac arrest before it could be initiated. An autopsy was performed, which revealed coagulative necrosis in the left anterograde-lateral wall, acute inflammatory cell infiltrate, and contraction band necrosis consistent with an acute myocardial infarction of an estimated 18- to 24-hour duration.

The results of the ADAMTS13 assay reported later showed her ADAMTS13 activity to be below 3% (normal: 68-163%), thereby confirming her diagnosis of TTP.

## Discussion

TTP is a thrombotic microangiopathy (TMA) caused by reduced activity of the vWF-cleaving protease ADAMTS13. It is a medical emergency that is almost always fatal if appropriate treatment is not initiated promptly [5].

TTP is a difficult diagnosis to make because of its diverse array of clinical manifestations, symptomatic overlap with other TMAs, and the limited availability of ADAMTS13 testing [6]. The classic symptoms of TTP are the pentad of fever, thrombocytopenia, MAHA, neurologic abnormalities, and renal failure [1]. It has, however, come to light that this symptomatic pentad is neither sensitive nor specific, as the majority of patients diagnosed with TTP do not experience all five clinical features [7].

Further complicating the clinical picture of TTP is the variable temporal onset of MAHA. Previously documented cases of atypical TTP have been characterized by the onset of MAHA later in the disease course, as was seen in our patient [8]. These cases serve as an important reminder that TTP cannot be ruled out as a potential diagnosis by the absence of schistocytes on the initial peripheral blood smear, as it is possible to have TTP with a delayed onset of MAHA.

It can be categorized as congenital or acquired, and many cases are idiopathic. However, many case reports have suggested an association with autoimmune diseases [9], infection [10], pregnancy [11], bone marrow transplantation [12], and certain drugs such as cyclosporine A, tacrolimus, mitomycin, or ticlopidine [13], which were not suggested in this patient's history. Also, in the literature, atypical TTP has been described in patients presenting with strokes or myocardial infarction with normal or near-normal laboratory values [14,15].

Also, further adding to the confusion was that our patient was an older individual, and TTP is rare in the elderly population. It usually affects people in the age group of 20 to 50 years. As seen in a recent study, where researchers evaluated data from 411 patients to determine disease characteristics particular to older patients, 71 were 60 years or older. Mortality was higher among older patients, with 26 of the 71 elderly patients dying within one month compared with 32 of the 340 younger patients [16].

The differential diagnosis includes Shiga toxin-producing Escherichia coli hemolytic uremic syndrome (STEC-HUS), atypical hemolytic uremic syndrome (aHUS), Evans syndrome, anti-phospholipid syndrome, disseminated intravascular coagulation (DIC), and other causes of TMAs including malignant hypertension, drugs, and disseminated cancer [4]. ITP is a diagnosis of exclusion and should not be made until other causes of thrombocytopenia are appropriately excluded [17]. Our patient was misdiagnosed with ITP on presentation. It is important to avoid conflating the diagnosis of ITP with “thrombocytopenia of unknown origin,” as ITP is a distinct clinical entity caused by an acquired thrombocytopenia caused by immune destruction of platelets [18].

In the literature also, there is a 7.5-day delay for the clinical diagnosis; hence, delay in the treatment is due to rarity of this condition. Furthermore, the delay in diagnosis and treatment is associated with a worse outcome [19]. There are prognostic scores developed to mitigate and reduce the chance of mistakes and increase the accuracy of clinical diagnosis. According to validation studies, the PLASMIC score has been shown to be practical and effective [20].

Other scores are the French score and those by Bentley et al. [20]. The advantage of the PLASMIC score over these scores is the simplicity and availability of laboratory scores since the other scores need extra evaluation, which is not always available [20].

Treatment guidelines for ITP are well established and include the use of glucocorticoids or IV immunoglobulin, depending on the patient’s presentation. The treatment for TTP is plasma exchange therapy. Although ITP treatments do not exacerbate TTP, they are ineffective at treating the underlying cause of disease in patients with TTP [2].

Current guidelines for TTP recommend that in the presence of a clinical history of fever, neurologic or abdominal symptoms, in addition to laboratory findings of microangiopathic hemolytic anemia, a schistocyte count of >1%, thrombocytopenia, or renal dysfunction and an absence of abnormal coagulation parameters, a tentative diagnosis of idiopathic TTP or acute TMA can be made, and clinicians can plan to initiate plasma exchange [19]. It should not be delayed until ADAMTS13 activity dosage results are available, as this may take several days in most centers.

Tests for ADAMTS13, its inhibitor, and ultra-large VWF levels should also be sent out in patients meeting the above criteria. It is important to utilize these broader guidelines when considering the initiation of plasma exchange therapy because, as seen in our patient, TTP can present in a variety of different ways. Empiric plasma exchange therapy has led to a dramatic improvement in TTP survivorship, whereas a delay in plasma exchange therapy is associated with poor outcomes [8]. With plasma exchange therapy, the survival rate of TTP is as high as 80-90% [19]. As a medical intervention essential to the patient’s survival, it is imperative that plasma exchange therapy is initiated as early as possible in a patient with suspected TTP.

## Conclusions

Our patient’s case presents an opportunity to appreciate the differences and overlap between diseases characterized by thrombocytopenia, hemolytic anemia, and/or TMA. Primarily, it demonstrates the importance of maintaining a wide differential diagnosis when a patient presents with profound thrombocytopenia. Furthermore, this case serves as a caution against anchoring to ITP as the cause of thrombocytopenia early in a patient’s admission. The history of use of antibiotics skewed the differential to ITP and ultimately delaying the diagnosis of TTP, leading to an inevitable and devastating consequence.

Overall, it is an example of one of the many ways in which TTP can present, and emphasizes the importance of considering TTP as a differential diagnosis even when the presentation is not "typical".
